# Phylogenetic and Recombination Analysis of Tomato Spotted Wilt Virus

**DOI:** 10.1371/journal.pone.0063380

**Published:** 2013-05-17

**Authors:** Sen Lian, Jong-Seung Lee, Won Kyong Cho, Jisuk Yu, Mi-Kyeong Kim, Hong-Soo Choi, Kook-Hyung Kim

**Affiliations:** 1 Department of Agricultural Biotechnology and Plant Genomics and Breeding Institute, Seoul National University, Seoul, Republic of Korea; 2 Institute for Agriculture and Life Sciences, Seoul National University, Seoul, Republic of Korea; 3 Department of Agricultural Biology, National Academy of Agriculture Sciences, Suwon, Republic of Korea; Institute of Infectious Disease and Molecular Medicine, South Africa

## Abstract

*Tomato spotted wilt virus* (TSWV) severely damages and reduces the yield of many economically important plants worldwide. In this study, we determined the whole-genome sequences of 10 TSWV isolates recently identified from various regions and hosts in Korea. Phylogenetic analysis of these 10 isolates as well as the three previously sequenced isolates indicated that the 13 Korean TSWV isolates could be divided into two groups reflecting either two different origins or divergences of Korean TSWV isolates. In addition, the complete nucleotide sequences for the 13 Korean TSWV isolates along with previously sequenced TSWV RNA segments from Korea and other countries were subjected to phylogenetic and recombination analysis. The phylogenetic analysis indicated that both the RNA L and RNA M segments of most Korean isolates might have originated in Western Europe and North America but that the RNA S segments for all Korean isolates might have originated in China and Japan. Recombination analysis identified a total of 12 recombination events among all isolates and segments and five recombination events among the 13 Korea isolates; among the five recombinants from Korea, three contained the whole RNA L segment, suggesting reassortment rather than recombination. Our analyses provide evidence that both recombination and reassortment have contributed to the molecular diversity of TSWV.

## Introduction

Tomato spotted wilt disease was first described in 1919 in Australia and was later identified as a viral disease caused by *Tomato spotted wilt virus* (TSWV) [1]. TSWV has been a common virus in Western countries (i.e., Western Europe and the USA) [2]–[4] and has gradually spread worldwide to temperate, subtropical, and tropical regions [5]. TSWV has recently been detected in the Middle East and far eastern Asia [6]. TSWV is vectored by a number of thrips species but most efficiently by the western flower thrips *Frankliniella occidentalis*, and the spread of *F*. *occidentalis* has contributed to the worldwide occurrence of TSWV and tomato spotted wilt disease [7].

TSWV causes serious damage to a wide range of economically important plants, and especially to vegetables such as tomatoes and potatoes [6], [8], [9]. The disease symptoms caused by TSWV are diverse and include ringspots, black streaks on petioles or stems, necrotic leaf spots, and tip dieback [10]–[14].

TSWV is assigned to the genus *Tospovirus* in the family *Bunyaviridae* [15]. In general, TSWV is transmitted in a persistent manner by certain thrips (Thysanoptera) species [16], [17]. The TSWV virion ranges in size from 80 to 120 nm and has a membrane-bound spherical structure [9]. TSWV consists of three single-stranded RNA segments, which are named L (8.9 kb), M (4.8 kb), and S (2.9 kb) based on their size [9]. The L RNA is a negative-stranded RNA that encodes the RNA-dependent RNA polymerase (RdRp), which plays an important role in viral replication [18]. In contrast, both M and S segments are ambisense RNAs with two open reading frames (ORFs), which are encoded from the viral sense and complementary strands [19], [20]. The M RNA encodes a Gn-Gc glycoprotein and a nonstructural protein (NSm) required for viral cell-to-cell movement [21]–[23]. S RNA encodes a nonstructural protein (NSs) involved in the suppression of gene silencing and the nucleocapsid (N) protein [24].

The whole-genome sequences have been determined for five TSWV isolates including three from Korea, one from China, and one from Brazil [25], [26]. Based on the large number of partial sequences for diverse TSWV isolates, several studies have addressed the issue of the origin of various TSWV isolates [27], [28].

Recombination, which refers to the formation of chimeric molecules from parental genomes of mixed origin and which can be important for evolution, commonly occurs with RNA viruses [29]. The exchange of genetic information between viruses with nonsegmented RNAs is termed RNA recombination. The exchange of genetic information between viruses with segmented RNAs, in which entire segments are exchanged, is termed reassortment [29]. Although RNA recombination and reassortment are mechanistically very different, both require that two or more viruses or different isolates of one virus infect the same host cell [28]–[30].

In this study, we determined the whole sequences for 10 TSWV isolates identified from various regions and hosts in Korea. In addition, we used these whole sequences plus available sequences for other TSWV isolates to study the molecular diversity and recombination events among TSWV isolates.

## Materials and Methods

### Whole-genome Sequencing of 10 TSWV Isolates

During 2009 and 2010, pepper, *Stellaria aquatica*, *Stellaria media*, *Lactuca indica*, lettuce, tomato, and chrysanthemum plants with typical symptoms of TSWV infection were collected from several geographical regions in Korea (Table 1). Ten new TSWV isolates (K4–K8, K10, K12, K16, K17, K18) were obtained.

**Table 1:** Geographic origin, isolation host, and genome segment size characteristics of the 13 Korean isolates of TSWV examined in this study.

Isolate	State	City	Host	Size (nt)			
				RNA L	RNA M	RNA S	Full-length
K1 (Tomato NJ-JN)	Jeollanam-do	Naju	Tomato	8913	4783	2968	16664
K2 (Pepper1 CY-CN)	Chungcheongnam-do	Chungyang	Pepper	8914	4768	3013	16695
K3 (Pepper2 CY-CN)	Chungcheongnam-do	Chungyang	Pepper	8914	4768	3013	16695
K4	Kyungsangbuk-do	Kimhae	Pepper	8913	4781	2971	16665
K5	Chungcheongnam-do	Yesan	*Stellaria aquatica*	8913	4792	2975	16680
K6	Jeollanam-do	Naju	*Stellaria media*	8913	4786	2969	16668
K7	Jeollanam-do	Naju	Pepper	8913	4785	2967	16665
K8	Chungcheongnam-do	Yesan	*Lactuca indica*	8913	4787	2977	16677
K10	Chungcheongnam-do	Yesan	*Stellaria aquatica*	8913	4791	2975	16679
K12	Jeollabuk-do	Namwon	Lettuce	8914	4829	2961	16704
K16	Chungcheongnam-do	Seosan	Tomato	8913	4788	2973	16674
K17	Jeollabuk-do	Namwon	*Stellaria media*	8914	4828	2961	16703
K18	Jeollanam-do	Yeongkwang	Chrysanthemum	8914	4770	3020	16704

cDNA synthesis, RT-PCR, cloning, and sequencing were performed as described previously [26]. In brief, total RNAs were extracted from the leaves of infected plants using TRI Reagent (Molecular Research Center, Inc., Cincinnati, USA) according to the manufacturer’s instructions. For cDNA synthesis, total RNAs with random primers and dNTPs were incubated at 65°C for 5 min and then incubated at 42°C for 1 h after M-MLV reverse transcriptase (RT) and M-MLV 5X buffer (Promega, USA) were added. For amplification of PCR fragments from individual cDNAs, 2 µl of cDNA was used as template. a total of 50 µl of cDNA reaction mixture including Ex Taq (Takara, Japan) was subjected to PCR for 35 cycles with an initial denaturation at 94°C for 10 min, 35 cycles of 30 s at 94°C for denaturation, 30 s at 53°C for annealing and 30 s at 72°C for extension, and a final extension 10 min at 72°C using a thermal cycler (My Cycler™, Bio-Rad). The QIAquick PCR purification kit (QIAGEN, Hilden, Germany) and pGEM-T Easy Vector (Promega, Madison, USA) were used for PCR product purification and for cloning of the purified PCR product, respectively. Sequencing of all clones was conducted at least three times in both orientations using universal and specific PCR primers at the National Instrumentation Center for Environmental Management (NICEM), Seoul National University, Korea. The primer sets were the same as previously used for sequencing the complete TSWV genomes of three isolates from Korea [26]. The previously published whole-genome sequences of the three TSWV isolates from Korea were included in this study, giving a total of 13 whole-genome sequences of TSWV from Korea.

### Sequence Assembly and Alignment

The obtained sequences were first analyzed with the MegAlign program implemented in the DNAStar 5.01 package (DNASTAR, Madison, USA). For the detailed sequence analysis or conversion of sequence file formats (http://www.nrbsc.org/gfx/genedoc/), the BioEdit sequence alignment editor (Version 7.0.9) (http://www.mbio.ncsu.edu/bioedit/bioedit.html) and GeneDoc programs were used. After sequence assembly, the complete sequences of each RNA segment were deposited in GenBank with accession numbers from KC261947 to KC261976.

For phylogenetic and recombination analysis of TWSV, complete RNA sequences for known TSWV isolates were retrieved from the GenBank in National Center for Biotechnology Information (NCBI). Detailed information for RNA sequences of each TSWV isolate is provided in Table S1. Nucleotide sequences were aligned using CLUSTALW implemented in MEGA 5.05 followed by manual modification [31].

### Construction of Phylogenetic Trees

Aligned nucleotide sequences were used to construct phylogenetic trees using the MEGA 5.05 software package [31]. Full-length sequences of TSWV RNAs L, M, and S segments were aligned and manually adjusted using CLUSTALW [32]. Phylogenetic trees were constructed based on the neighbor-joining (NJ) method and Kimura 2-parameter method. Bootstrap resampling (1000 replications) was used to measure the reliability of individual nodes in each phylogenetic tree.

### Network Analysis Using Splitstree

Splits networks were created with SplitsTree 4.11 [33], using the uncorrected P characters transformation. For network creation, NEXUS format files were generated from MEGA 5.05 software based on the aligned RNA segment sequences.

### Recombination Analysis Using RDP4

The Recombination Detection Program v.4.16 (RDP4) was used for recombination analysis. Recombination events, likely parental isolates of recombinants, and recombination break points were analyzed using the RDP, GENECONV, Chimaera, MaxChi, BOOTSCAN, and SISCAN methods implemented in the RDP4 program with default settings [34].

## Results

### Whole-genome Sequencing for 10 Korean TSWV Isolates

From 2009 to 2010, we obtained a total of 18 TSWV isolates from plant samples with TSWV disease symptoms that were collected from various geographical regions in Korea. Of them, the 10 isolates were obtained from seven host plants, including pepper, *Stellaria aquatica*, *Stellaria media*, *Lactuca indica*, lettuce, tomato, and chrysanthemum (Table 1). We then determined the whole-genome sequences for these new TSWV isolates. The GenBank accession numbers of the TSWV RNA segments are listed in Table S1. The sizes of RNA L (8913 or 8914 bp) were not variable, while the sizes of RNA M (4770 to 4829 bp) and RNA S (2961 to 3020 bp) were variable (Table 1). Isolates K12 from lettuce and K18 from chrysanthemum were the largest (16704 bp) while isolates K4 and K7 from pepper were the smallest (16665 bp) (Table 1). When the previously reported whole-genome sequences of the three TSWV isolates from Korean (K1–K3) [26] were included in the analysis, we compared the nucleotide sequence identity among the 13 Korean TSWV isolates ranging from 93.6 to 99.9% (Table 2). Sequence identity was highest between isolates K2 and K3 and lowest between isolates K2 and K12. In addition, we compared the nucleotide identity among 13 the Korean TSWV isolates based on each RNA segment sequence (Table S2). The lowest nucleotide sequence identities of RNA M and RNA S among the 13 isolates were 97.9% and 96.7%, respectively. However, the lowest nucleotide sequence identity of RNA L among the 13 isolates was only 79% displaying strong genetic diversity of the RNA L nucleotide sequences as compared to those of RNA M and RNA S. In particular, the RNA L nucleotide sequences of three isolates (K2, K3, and K13) were very different from other 10 Korean isolates. Interestingly, the whole genome size of the 13 isolates differs by a maximum of 39 nt whereas the individual RNA M and RNA S differ by up to 59 nt. Thus, it appears possible that the largest RNA S may not be compatible with the largest representatives of RNA M, and the smallest RNA S may not be compatible with the smallest representatives of RNA M. It would be surprising if there were such tight control over the total size of the genome that could be encapsidated.

**Table 2:** Nucleotide sequence similarity matrix index for the 13 Korean isolates of TSWV based on the full genome sequence.

	K1	K2	K3	K4	K5	K6	K7	K8	K9	K10	K11	K12	K13
**K1**		94.6	94.6	98.9	99.4	99.7	99.7	99.5	99.5	96.3	99.5	96.4	94.6
**K2**	5.4		99.9	94.6	94.6	94.6	94.6	94.6	94.6	93.7	94.6	93.6	99.6
**K3**	5.4	0.1		94.6	94.6	94.6	94.6	94.6	94.6	93.6	94.6	93.7	99.6
**K4**	1.1	5.4	5.4		98.8	98.9	98.9	98.9	98.9	96.3	98.8	96.3	94.6
**K5**	0.6	5.4	5.4	1.2		99.4	99.4	99.7	99.7	96.3	99.6	96.3	94.5
**K6**	0.3	5.4	5.4	1.1	0.6		99.7	99.5	99.5	96.3	99.5	96.3	94.6
**K7**	0.3	5.4	5.4	1.1	0.6	0.3		99.5	99.5	96.4	99.4	96.4	94.6
**K8**	0.5	5.4	5.4	1.1	0.3	0.5	0.5		99.8	96.4	99.7	96.3	94.6
**K9**	0.5	5.4	5.4	1.1	0.3	0.5	0.5	0.2		96.3	99.6	96.3	94.6
**K10**	3.7	6.4	6.4	3.7	3.7	3.7	3.6	3.6	3.7		96.3	99.7	93.6
**K11**	0.6	5.4	5.4	1.2	0.4	0.6	0.6	0.3	0.4	3.7		96.3	94.5
**K12**	3.7	6.4	6.3	3.7	3.7	3.7	3.7	3.7	3.7	0.3	3.7		93.6
**K13**	5.4	0.5	0.4	5.4	5.5	5.4	5.4	5.4	5.4	6.4	5.5	6.4	

The values were calculated using MEGA 5. Values above and below the diagonal shaded frames indicate the percentage of nucleotide sequence identity and divergence, respectively.

### Phylogenetic Relationships of TSWV Isolates

**Figure 1:**
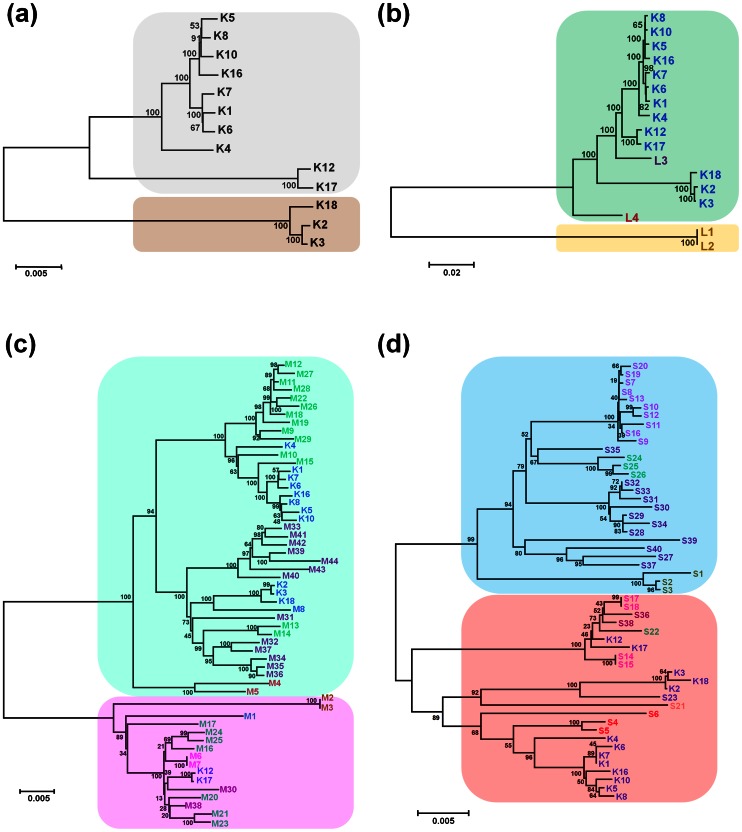
Phylogenetic trees of full-length TSWV isolates and RNA segments. (a) A phylogenetic tree of the 13 Korean TSWV isolates, including 10 that were newly sequenced in this study and three that were previously reported [26]. The phylogenetic tree was constructed by the neighbor-joining (NJ) method and Kimura 2-parameter method with bootstrap resampling (1000 replications) using the whole-nucleotide sequences by combining RNA L, RNA M, and RNA S sequences. (b) A phylogenetic tree of 17 full-length TSWV RNA L segments. L1 and L2 were from Brazil whereas L3 and L4 were from the USA and China. Note that in (b–d), the country where each TSWV isolate/segment was collected is indicated by different-colored fonts. (c) A phylogenetic tree of 57 full-length TSWV RNA M segments. M1 was from Australia, M2 and M3 were from Brazil, M4 and M5 were from China, M6 and M7 were from Italy, M8 was from Korea, M9 to M29 were from Spain, and M30 to M44 were from the USA. The remaining 13 isolates were from Korea. (d) A phylogenetic tree of 53 full-length TSWV RNA S segments. S1–S3 were from Brazil; S4–S6 were from China; S7–S20 were from Italy; S21–S23 were from Japan, the Netherlands, and Korea, respectively; S24–S26 were from Spain, and S27–S40 were from the USA. The remaining 13 isolates were from Korea. Detailed information for each TSWV isolate is also provided in Table 1 and Table S1. The number at each branch of each phylogenetic tree represents the bootstrap value (1000 replicates). The different background colors indicate the different clusters of TSWV isolates.

To determine the genetic relationships among the 13 Korean TSWV isolates, we constructed a phylogenetic tree using the whole-nucleotide sequences after combining sequences of RNA L, RNA M, and RNA S. The analysis divided the 13 isolates into two groups (Figure 1a). The first group contained two sub-groups, one of which included four isolates from Chungcheongnam-do (K5, K8, K10, and K16), three isolates from Jeollanam-do (K1, K6, and K7), and one isolate from Kyungsangbuk-do (K4); the second sub-group included two isolates from Namwon in Jeollanam-do (K12 and K17). The second group included two isolates from Chungyang in Chungcheongnam-do (K2 and K3) and one isolate from Yeongkwang in Jeollanam-do (K18).

To compare the phylogenetic relationships of the Korean TSWV isolates with the other known TSWV isolates, we obtained complete sequences for TSWV RNA segments from GenBank and then constructed phylogenetic trees for each segment. We obtained full-length sequences for the known other TSWV isolates including seven (RNA L), 47 (RNA M), and 43 (RNA S) segments from GenBank in NCBI (Table S1). Those full-length TSWV RNA segments were obtained from nine countries including Australia, Brazil, China, Italy, Japan, the Netherlands, South Korea, Spain, and the USA. The host ranges associated with these sequences were diverse and included aster, buttercup, chrysanthemum, dahlia, *Emilia sonchifolia*, *Falso lulo*, *Lactuca sativa*, peanut, pepper, tobacco, tomato, and several unknown host plants (Table S1).

Note that in the trees displayed in Figure 1b–d, the color of the font used for isolate designation is different depending on the country of origin. The phylogenetic tree based on 17 RNA L segments had two groups (Figure 1b). All 13 isolates from Korea together with one isolate from China and one from the USA formed one group, while two isolates from Brazil formed the second group (Figure 1b). The first group could be divided into two sub-groups; one included only one isolate from China and the other included all 13 isolates from Korea and one from the USA. These results, which are based on whole RNA L sequences, suggest that two isolates from Brazil (L1 and L2) are very different from the other TSWV isolates.

The phylogenetic tree based on 57 RNA M segments had two well-defined groups (Figure 1c). The first group was further divided into two sub-groups; one of these contained 12 isolates from Spain and eight from Korea while the other contained 13 isolates from the USA, four from Korea, and two from Spain. Interestingly, two isolates from China formed another sub-group in the first group, and these two isolates were separated from the other 39 isolates in the first group, which were derived from Korea, the USA, and Spain, by a substantial genetic distance. It appears that the TSWV isolates in the first group were clustered according to their geographical regions. In the second group, two isolates from Brazil (M2 and M3) were clustered into one sub-group while the other 14 isolates (from Spain, Korea, the USA, Italy, and Australia) were clustered in a second sub-group.

The phylogenetic tree based on 53 RNA S segments also had two groups (Figure 1d). The 28 isolates in the first group included those from Italy, the USA, Spain, and Brazil, and those isolates were further divided into several sub-groups based on geographical region. The second group, which contained all 13 isolates from Korea, was further divided into two subgroups. One subgroup contained two isolates from Korea (K12 and K17) and seven isolates from Italy, the USA, and the Netherlands. The second sub-group contained only isolates from Asian countries, i.e., 12 isolates from Korea including S23, three isolates from China, and one isolate from Japan.

### Phylogenetic Networks of TSWV Isolates

**Figure 2:**
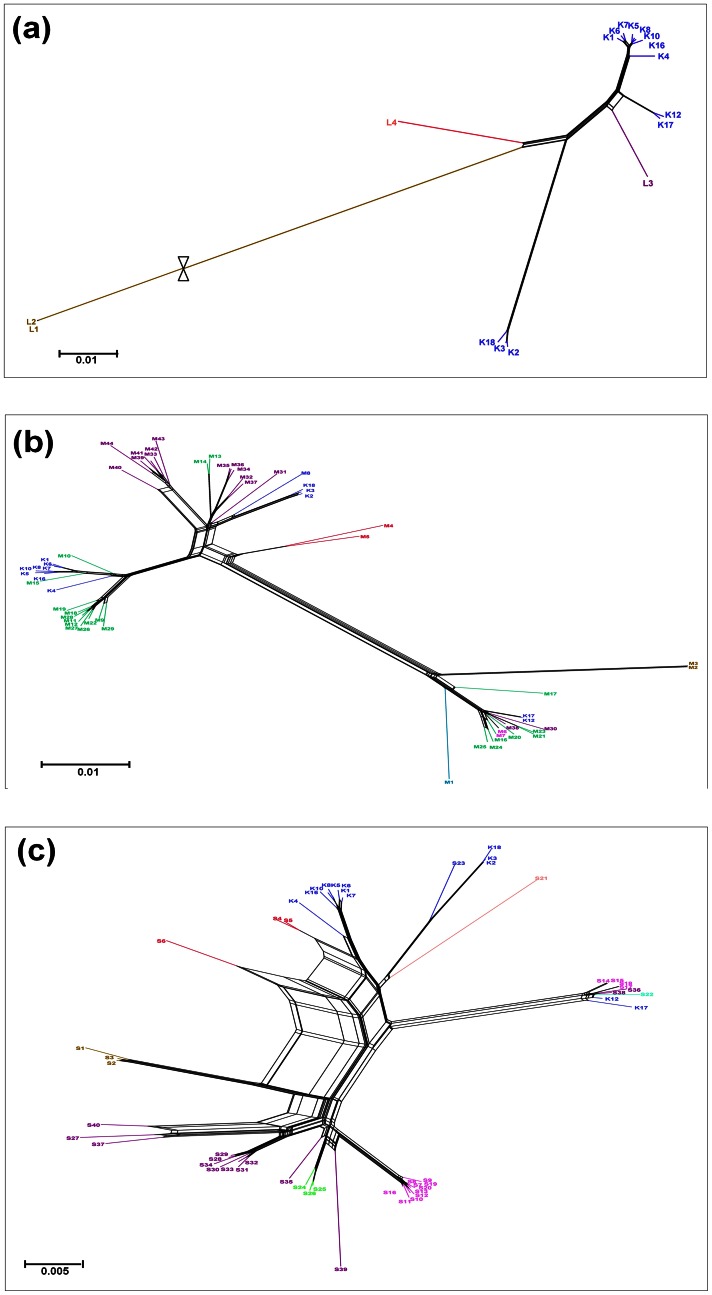
Phylogenetic networks of full-length TSWV RNA segments generated by Splitstree. (a) A splitstree network of 17 full-length TSWV RNA L segments. (b) A splitstree network of 57 full-length TSWV RNA M segments. (c) A splitstree network of 53 full-length TSWV RNA S. The country in which each segment was collected is indicated by a different color, such as blue indicates Korea, light blue indicates Australia, light brown indicates Brazil, red indicates China, pink indicates Italy, light green indicates Spain, purple indicates USA, light pink indicates Japan, and sky blue indicates Netherlands. Detailed information for each TSWV isolate is also provided in Table 1 and Table S1. The networks were created with SplitsTree 4.11 using the uncorrected P characters transformation.

Using the SplitsTree program, we generated three splits networks based on the complete sequences of RNA L, M, and S in order to elucidate recombination networks among the examined TSWV isolates. All three splits networks displayed reticulate topologies indicating several potential recombination events among the TSWV isolates (Figure 2). The Splitstree network based on RNA L is less complex than those based on RNA M and RNA S because of the limited number of isolates. For RNA L and RNA M, the network groups are highly consistent with the groups defined by the phylogenetic trees (Figure 2a and 2b). The splitstree of RNA L exhibited three parallel branches indicating that recombination might have occurred among three TSWV isolate groups in Korea (Figure 2a). The splitstrees of RNA M (Figure 2b) and S (Figure 2c) suggest that recombination might have occurred among the isolates collected from the same geographical regions, such as among the 10 isolates from Spain and the seven isolates from the USA (Figure 2b). The splitstree of RNA M and S, however, also indicate that several recombination events might have occurred for RNA M and S among isolates collected from different countries.

### Recombination Analysis of TSWV Using RDP4

**Figure 3:**
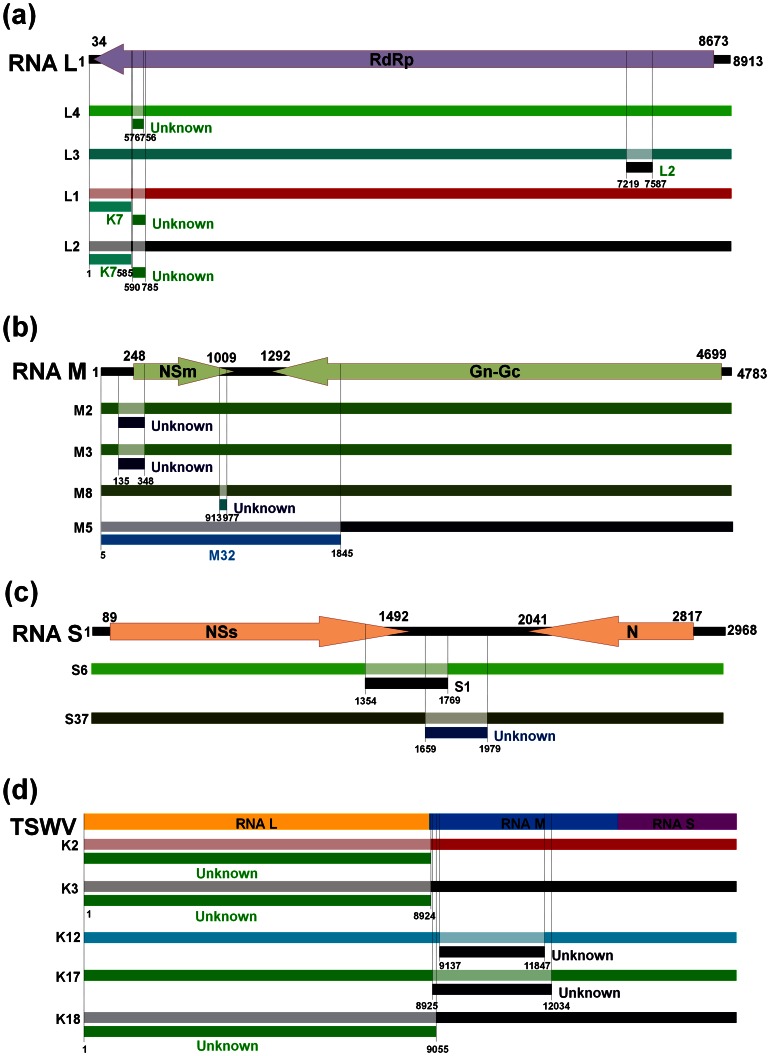
Analysis of possible recombination in full-length segments of TSWV RNA L, RNA M, and RNA S and in full-length sequences of 13 TWSV isolates from Korea. (a) Four recombination events were detected in full-length RNA L segments L1 and L2 from Brazil, L3 from USA, and L4 from China. (b) Four recombination events were detected in full-length RNA M segments M2 and M3 from Brazil, M5 from China, and M8 from Korea. (c) Two recombination events were detected in full-length RNA S segments S2 from China and S37 from the USA. Detailed information for each TSWV isolate/segment is also provided in Table 3 and Table S1. Each RNA segment is indicated by different color bar.

To further define the putative recombination signals observed by the network analysis, we performed recombination detection analysis using the RDP4 program, which contains various recombination detection algorithms like RDP, GENECONV, Chimaera, MaxChi, BOOTSCAN, and SISCAN. Using these six algorithms, we detected at least six recombination events in the 17 RNA L segments (Table 3 and Figure 3). Both RDP and Bootscan predicted six events while GENECONV did not detect any event. Events 1 to 3 were detected by RDP, Bootscan, Maxchi, and Chimaera. Segment L1 and L2 were predicted to have had two recombination events, which were localized from 1 bp to 585 bp and from 590 bp to 785 bp, respectively, in the RNA L (Figure 3a). Segments L3 and L4 seem to carry one recombinant event (from 7219 bp to 7587 bp for L3, and from 576 bp to 756 bp for L4). K1 is parental to L1, L2, and L4, with L1 and L2 possibly derived from L4. We found that there were similar breakpoints in different recombinants such as L1, L2, and L4 (Figure 3a). This might result from sequential recombination from an initial recombinant parent. Recombination analysis detected four recombinant events in the 47 RNA M segments including M2, M3, M5, and M8 with a high degree of reliability (Table 3 and Figure 3b). The recombinant events for both M2 and M3 were localized from 135 bp to 348 bp with M1 as a major parent. The predicted recombination parents for M5 and M8 were M4 and K18, respectively. In the case of RNA S, only two recombinant events were identified among the 53 segments (Figure 3c). The first recombination event in S6 predicted by all six algorithms was localized from 1354 bp to 1769 bp with S23 as a parent. The second recombination event in S37 was predicted by only GENECONV and SiSscan and was localized from 1659 bp to 1979 bp with S39 as a parent.

**Table 3:** Summary of unique recombination events identified by the Recombination Detection Program v.4.16 (RDP4).

		Breakpoint position in recombinant sequence			Parental sequence(s)		Score for the six detection methods in RDP4					
RNA segment	Event number	Begin	End	Recombinant Sequence(s)	Minor	Major	RDP	GENECONV	Bootscan	Maxchi	Chimaera	SiSscan
RNA L	1 (7)^a^	590	785	L1	Unknown (K3)	K1	1.99E-02	NS	1.56E-02	1.74E-02	6.30E-02	NS^b^
	2 (7)	590	785	L2	Unknown (K3)	K1	1.99E-02	NS	1.56E-02	1.74E-02	6.30E-02	NS
	3 (7)	576	756	L4	Unknown (K3)	K1	1.99E-02	NS	1.56E-02	1.74E-02	6.30E-02	NS
	4 (8)	7219	7587	L3	L2	K7	1.16E-02	NS	8.63E-03	NS	NS	NS
	5 (9)	1	585	L1	K7	L4	2.59E-02	NS	4.86E-02	NS	NS	2.48E-05
	6 (9)	1	585	L2	K7	L4	2.59E-02	NS	4.86E-02	NS	NS	2.48E-05
RNA M	1 (1)	135	348	M2	Unknown (K10)	M1	1.94E-24	6.33E-22	2.49E-18	4.13E-09	2.18E-08	NS
	2 (1)	135	348	M3	Unknown (K10)	M1	1.94E-24	6.33E-22	2.49E-18	4.13E-09	2.18E-08	NS
	3 (2)	5	1845	M5	M32	M4	3.46E-07	NS	3.90E-03	7.67E-13	6.67E-11	1.65E-03
	4 (3)	913	977	M8	Unknown (M21)	K18	1.03E-10	1.43E-09	4.62E-08	1.08E-02	2.28E-02	NS
RNA S	1 (1)	1354	1769	S6	S1	S23	8.50E-09	8.25E-08	3.50E-08	1.55E-05	4.83E-06	2.64E-11
	2 (4)	1659	1979	S37	Unknown (S11)	S39	NS	1.20E-03	NS	NS	NS	2.69E-05
13 Korean isolates of TSWV based on the full genome sequence	1 (1)	1	8924	K2	Unknown (K17)	K5	1.56E-26	NS	8.04E-26	1.29E-20	1.15E-24	1.15E-62
	2 (1)	1	8924	K3	Unknown (K17)	K5	1.56E-26	NS	8.04E-26	1.29E-20	1.15E-24	1.15E-62
	3 (1)	1	9055	K18	Unknown (K17)	K5	1.56E-26	NS	8.04E-26	1.29E-20	1.15E-24	1.15E-62
	4 (3)	9137	11847	K12	Unknown (K18)	K5	NS	NS	NS	9.28E-03	9.32E-04	1.79E-25
	5 (3)	8925	12034	K17	Unknown (K18)	K5	NS	NS	NS	9.28E-03	9.32E-04	1.79E-25

Foot note: a: Recombination event indicator; b: Not significant.

To investigate whether recombination events might have occurred among the 13 TSWV isolates from Korea, which was suggested by the network analysis, we performed recombination detection using only the whole sequences for the 13 TSWV isolates from Korea. As expected, there were at least five recombination events in isolates K2, K3, K12, K17, and K18 (Table 3 and Figure 3d). Interestingly, isolate K5 was regarded as a potential parent for five recombination events. The breakpoint position for isolates K2, K3, and K18 was initiated from the first bp in RNA L and ended at different positions that were mostly localized at the 5′ end of RNA M. These data suggested that the entire RNA L sequences for isolates K2, K3, and K18 might have been obtained from isolate K17 by reassortment. The other two recombinant positions were localized from 9137 bp to 11847 bp for K12 and from 8925 bp to 12034 bp for K17 indicating recombination events within RNA M.

## Discussion

In spite of the importance of TSWV as a re-emerging plant virus, only a limited number of complete genome sequences for TSWV are available. In this study, we determined the whole-genome sequences for 10 TSWV isolates collected from various hosts in Korea. To elucidate the molecular diversity of TSWV isolates in Korea, we analyzed these 10 TSWV isolates plus three others previously reported from Korea [26]. Although comparative analysis of whole-genome sequences indicated that TSWV isolates in Korea are highly conserved, the nucleotide sequence identity of RNA L segment among the 13 Korean isolates was very low and phylogenetic analysis revealed two groups. Interestingly, the largest group of TSWV isolates in Korea could be further divided into several sub-groups. This suggested genetic divergence of Korean TSWV isolates in RNA L nucleotide sequence containing RdRp in spite of their relatively high conservancy in RNA M and RNA S nucleotide sequences. Similarly, several studies also reported that strong genetic variability and recombination events could be identified in the RdRp region of various plant viruses [35]–[38]. Therefore, to study genetic diversity of Korean TSWV isolates, it is better to sequence a large number of RNA L segments than RNA M and RNA S segments. We performed phylogenetic analysis to find possible influence of the host of origin in Korean TSWV isolates. However, we found that sequence similarity among TSWV isolates depends more on geographical region in Korean than on host plant. A similar pattern has been observed for other plant viruses such as *Cucumber mosaic virus* (CMV) and *Beet curly top virus* [30], [39]. In addition, three isolates (K2, K3, and K18) in the second group of Korean isolates are very different from the other Korean isolates, indicating that TSWV in Korea might have been introduced more than once, might have diverged, or might have been altered in some cases by the arrival of new TSWV genes. The phylogenetic trees based on the 13 Korean TSWV isolates and other TSWV segments derived from other countries revealed the possible origin or ancestor of the Korean isolates. Phylogenetic analysis of RNA M segments demonstrated that Korean isolates in the largest group were closely related to the isolates from Spain, except that Korean isolates K12 and K17 (from Namwon, Jeollabuk-do) belonged to the other group along with several isolates from the USA, Spain, and Italy. In contrast, the phylogenetic analysis of RNA S segments suggested that most Korean isolates are related to the isolates from China and Japan rather than to the isolates from Western Europe or the USA. Moreover, only K12 and K17 clustered with a mixture of TSWV isolates from the USA, Spain, and Italy in the analysis based on RMA S, which is consistent with the result of RNA M. Similarly, the phylogenetic tree based on RNA L sequences confirmed that K12 and K17 were more closely related to an isolate from the USA than to those from China and Brazil. Overall, the phylogenetic data suggest that isolates K12 and K17 in Jeollabuk-do might have originated from Western Europe or the USA rather than from Asia.

Based on the phylogenetic information discussed in the previous paragraph, we hypothesize that the Korean TSWV isolates have two different origins, i.e., western countries (western Europe/USA) and Asian countries. Except for K12 and K17, the genetic relationships for most Korean TSWV isolates were consistent among the phylogenetic trees based on RNA L, RNA M, and RNA S segments. Our previous report also suggested that isolates K1, K2, and K3 might have originated from an Asian ancestor [26].

Genetic reassortment among TSWV isolates has been previously reported [28], [40]. For example, a previous study described genetic reassortment among three specific isolates which exchanged segments in a non-random manner, with the dominance of the TSWV-D RNA S over that of TSWV-10 presumed to be due to the presence of duplication in the intergenic region of TSWV-10 RNA S [40]. Another study reported that sequences in the intergenic region of TSWV RNA S correlated with geographic origin of the isolates examined and identified three conserved parts within the intergenic region [41]. In a specific condition, TSWV RNA S becomes dominant when the host plant is resistant to TSWV due to transgenic expression of N-protein [42]. The genomes of isolates K12 and K17 seem to consist of RNA segments that have different origins, i.e., RNA L and RNA M for K12 and K17 were predicted to be derived from non-Asian countries (western Europe/the USA) while RNA S was predicted to be derived from Asian countries. Thus, we suspect that the RNA S segments of TSWV isolates from western countries might have been displaced by those of isolates derived from China or Japan in evolutionary history. In general, the RNA S segment of TSWV has low competitive ability and can be easily displaced during virus evolution [28]. Therefore, displacement of one kind of RNA S segment might have required only a few recombination events; our recombination analysis identified only two recombination events for the RNA S segment, which was significantly fewer than for RNA L and RNA M segments. Such genome reassortment of RNA S segments is required for TSWV to overcome N gene-derived resistance in a new host plant [42], and the intergenic region (IGR) in RNA S might serve a regulatory function in genome reassortment [40].

Recombination analysis of TSWV sequences identified a total of 12 recombination events, and the number of recombination events may be positively related to the size of the RNA segment. An increased number of samples did not always result in the detection of an increased number of recombinants; for example, only two recombination events were detected based on a large number (53) of RNA S segments. If we select recombination events supported by at least three different methods of analysis and with P-values <1.0×10^−6^, all recombination events in RNA L would be discarded while all four recombination events in RNA M and one recombinant event in RNA S would be accepted. In the case of the 13 Korean TSWV isolates, the recombination events in K2, K3, and K18 would be accepted. Thus, the threshold for accepting the recombination prediction can strongly affect the result when the sample number is small, as is the case with RNA L in the current study.

Interestingly, most parents of detected TSWV recombinants originated from foreign countries. For instance, the major parent of M2 and M3 from Brazil was M1 from Australia, and the major parents of isolates M5 and S6 from China were isolates from the USA and Korea, respectively. In the analysis restricted to the 13 Korean isolates, isolate K5 was identified as the single parent for five recombinants, which provides evidence for recombination events that occurred only within Korea. We suspect, however, that many newly introduced TSWV isolates could recombine with local or other introduced isolates in order to adapt to new hosts or other new environmental conditions. We also detected recombination events after combining the three RNA segments of the 13 Korea isolates. Of the identified recombination events that were reliably predicted, positions for three recombinants included the entire RNA L segment, indicating that the identified three recombinants in RNA L might be the result of reassortment rather than recombination. Many studies demonstrated that reassortment plays an important role for the production of TSWV recombinants. For example, although a recent study identified 38 recombination isolates among 224 TSWV isolates, no recombination signal was detected within each of the four sequenced regions or between the NSs and N genes corresponding to RNA S; this suggested that the isolates were reassortments rather than recombinants [28].

For single-stranded RNA viruses, recombination is a major evolutionary way for an isolate to adapt to new environmental conditions and hosts. For example, frequent recombination was occurred in various *Soybean mosaic virus* and potyvirus isolates [43]–[45]. Moreover, reassortment frequently occurs in natural populations of animal viruses with segmented genomes [46], [47] and in natural populations of plant viruses with multipartite genomes [48], [49]. For multipartite RNA viruses, both recombination and reassortment can be important for genetic exchange [50]. Previous studies reported that genetic exchange by recombination or reassortment is infrequent in natural populations of CMV [51], [52]. In contrast, *Brome mosaic virus*, a tripartite virus, displayed frequent homologous recombination events [53]. Researchers still debate, however, whether recombination and reassortment is more important for the evolution of multi-segment RNA viruses. One hypothesis is that recombination does not occur or occurs very infrequently for multi-segment RNA viruses [54]–[56], while a second hypothesis is that both RNA reassortment and recombination contribute to genetic exchange [30]. The rates of recombination and reassortment may be associated with particular genome structures and viral life cycles [29]. In addition, the structure of the replication complex, which differs between viral groups, could determine whether recombination or reassortment predominates.

In the case of TSWV, previous reports have indicated that both recombination and reassortment occur in nature [28], [40], and the current study also provided evidence for both recombination and reassortment of TSWV isolates. According to our results, the rate of TSWV recombination in nature is significantly lower than that of reassortment. Both recombination and reassortment are presumably favored when an isolate is introduced into a new region where potential hosts may already be infected with a different isolate.

At least two questions remain. First: Why were only a few recombination events detected among the large numbers of TSWV RNA segments? Second: Why were no recombination events detected among the isolates from the same geographical region such as the USA and Spain although previous studies have used many RNA segments for the analysis. To answer these and other questions concerning the genetic diversity, origin, and mechanisms of genetic exchange for TSWV, researchers will need to obtain and study the whole-genome sequences for a large number of TSWV isolates collected from many different areas of the world. In parallel, an equally valid experimental approach would be to make mixed infections of isolates of different geographic origins, and to assay for recombination versus reassortment after passaging; by passaging in different hosts, it would also be possible to address the question of host influence on adaptation through recombination and reassortment.

## Supporting Information

Table S1 Detailed information for TSWV isolates and segments used in this study.
(XLS)

Table S2 Nucleotide sequence similarity matrix index among 13 Korean TSWV isolates based on RNA L (A), RNA M (B) and RNA S (C) sequences.
(XLS)
